# Structurally Different Pectic Oligosaccharides Produced from Apple Pomace and Their Biological Activity In Vitro

**DOI:** 10.3390/foods8090365

**Published:** 2019-08-26

**Authors:** Agnieszka Wilkowska, Adriana Nowak, Aneta Antczak-Chrobot, Ilona Motyl, Agata Czyżowska, Anna Paliwoda

**Affiliations:** 1 Institute of Fermentation Technology and Microbiology, Faculty of Biotechnology and Food Science, Łódź University of Technology, Wólczańska 171/173, 90-924 Łódź, Poland; 2 Institute of Technology and Analysis of Food, Faculty of Biotechnology and Food Science, Łódź University of Technology, Wólczańska 171/173, 90-924 Łódź, Poland

**Keywords:** pectin-derived oligosaccharides (POS), apple pomace, prebiotics, short chain fatty acid, simulation/inhibition of adhesion

## Abstract

This study set out to identify the composition and the biological activity of pectin-derived oligosaccharides (POS) generated from mild acid or enzymatic hydrolysis of apple pomace (AP). The effect of the polymerization of the structural units of POS contained in the AP hydrolysate on the growth and metabolism of microbiota from the human gastrointestinal tract and the adhesion of lactic acid bacteria (LAB) or pathogens to human gut epithelial cells was investigated in vitro. Mild acid hydrolysis followed by pectinolysis with Rohapect MaPlusT yielded the highest concentration of POS. In contrast, pure enzymatic processing of the AP performed with a mixed preparation of cellulase and Rohapect MaPlusT resulted in 1.8-fold lower overall POS. The concentration of higher-order oligosaccharides (degree of polymerization (DP) 7–10), however, was 1.7-fold higher. The increased ratio of higher-order oligosaccharides caused an increase in the bifidogenic effect, as well as affecting the amount and nature of short-chain fatty acid produced. Inhibition of *Enterobacteriaceae* was also observed. The strongest stimulation of LAB adhesion to the human epithelial cells occurred in the presence of the preparation containing the highest concentration of higher-order oligosaccharides. The fecal bacteria and pathogens showed much weaker adhesion to intestinal cells in the presence of all the tested AP hydrolysates. Both of the tested POS preparations, containing structurally different oligosaccharides (DPs 2–10 with different ratios of higher-order oligosaccharides), have the potential to be used as prebiotics for humans and animals. They stimulate bowel colonization with lactic acid bacteria and inhibit the development of infections caused by pathogens.

## 1. Introduction

Apples (*Malus* sp.) are one of the most intensely cultivated fruits in the world. In 2017, the production of apples (83.1 million tons) was exceeded only by that of watermelons (118.4 million tons) and bananas (113.9 million tons) [1]. Processing of about 50%–60% of apples into juice and juice concentrate raises the problem of waste management. Apple pomace (AP) is the main waste from technological apple processing, and accounts for 20% of the processed raw material. It is a valuable and recyclable waste, but still underexploited. Due to its being rich in carbohydrates, organic acids, fiber, and minerals, APs are considered a particularly valuable group of biodegradable natural products [2]. The management of AP over the last 20 years has changed significantly. In the past, it was used mainly for cattle feed. Nowadays, it is also used in food production: as pectin, fiber and texture additives for the production of nutritional breads, and as a functional spice in meat products. The development of enzymology has enabled the production of many valuable substances from AP, such as polyphenols, fiber, and prebiotics [3].

The Food and Agriculture Organization [4] defines prebiotics as unviable nutrients which have beneficial effects on the health of the host, due to the modulation of intestinal microbiota. The principle of prebiotics is based on the selective stimulation of intestinal microorganisms in the large intestine, which are able to hydrolyze prebiotics to monomers and to use them to grow. As a result, prebiotics are able to restrict the growth of many pathogenic bacteria in the gut, because the prebiotic ingredients allow them to grow only to a limited extent or not at all.

On an industrial scale, prebiotics are produced in enzymatic processes using hydrolases or transglycosylases, in chemical processes (catalytic saccharide conversion), or in a microbiological way. Prebiotic preparations can be manufactured from natural sources, such as lignocellulosic raw materials, lactose, starch, sucrose, inulin, xylan, or plant extracts (e.g., soy) [5,6]. Due to its low cost and ease of acquisition, AP can compete with other raw materials used for the manufacture of prebiotics, namely pectic oligosaccharides. Apple pomace is characterized by significant content of pectin and cellulose. The pectin content of dried AP has been found to be over 20.9% [7]. The latest research suggests that pectin-derived oligosaccharides (POS) are a promising source of next generation prebiotics.

Previous studies have focused on oligosaccharides generated from several pectin-rich feedstocks, including citrus peel, sugar beet pulp, and potato pulp [3,8,9,10,11]. The production of POS from AP by enzymatic means has been reported in References [12,13,14]. However, the experimental results only confirmed its bifidogenic effect by means of in vitro fermentability, using microbial strains or human fecal inocula [15]. There have been no studies on the influence of AP POS on the adhesion of bifidobacteria and pathogens to intestinal epithelial cells. Adhesion is an important process for the survival and proliferation of probiotic bacteria in the digestive tract. The first stage in the pathogenesis of many intestinal bacterial diseases is the adhesion of pathogens to epithelial cells of the intestine via specific sugar groups on their surfaces [16]. Prebiotic oligosaccharides introduced into the intestine with food may competitively inhibit the binding of bacteria to intestinal epithelial cells, reducing the frequency and severity of diseases of the digestive system.

The aim of the present study was to evaluate the potential of AP as a raw material for the production of POS by enzymatic and mild acid hydrolysis. Structurally different POS mixtures were examined as potential sources of prebiotics, in terms of their impact on the growth and metabolism of the microbiota of the human gastrointestinal tract and the adhesion to intestinal epithelial cells of lactic acid bacteria, fecal bacteria, and selected pathogens.

## 2. Materials and Methods

### 2.1. Materials

Apple pomace was obtained by pressing ground Cortland apples on a laboratory horizontal screw. The apples were bought at the local market and were cultivated during 2016.

Commercial enzymatic preparations were used: Rohapect Ma Plus T (AB Enzymes), Rohament CL (AB Enzymes), Pectinex Ultra SP-L (Novozymes), Viscozyme (Novozymes), Pectinex Ultra AFP (Novozymes), Rapidase Smart (DSM), and Cellulosoft 1.5 L (Novo Nordisk).

### 2.2. Mild Acid Hydrolysis Combined with Enzymatic Hydrolysis

The AP was diluted with 0.1 M HCl in the proportion 1:3 (*w/w*) and blended. The pH of the mixture was adjusted to 1.5 using concentrated HCl. Prepared samples were incubated in a water bath at 80 °C for 10 min and 30 min. After cooling, the samples were neutralized, and the pH was adjusted to 5.0 using 0.1 M NaOH.

After acid hydrolysis, pectinolytic preparations were added. The types of enzymes applied were polygalacturonases, pectin lyases, and cellulases. The enzymes were used at concentrations of 75 ppm. The samples were kept in a water bath at 40 °C for 10 min. Enzyme denaturation was performed at a temperature of 80 °C for 10 min.

### 2.3. Enzymatic Hydrolysis

**Figure 1:**
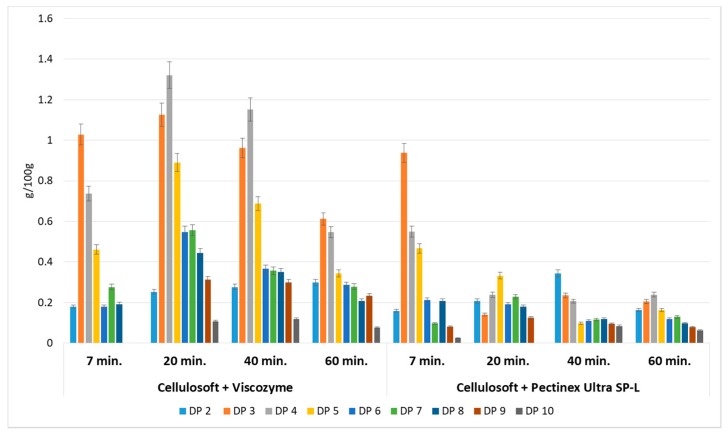
Yield of oligosaccharides with different degrees of polymerization obtained by enzymatic hydrolysis of apple pomace. Data represent mean from two independent experiments repeated three times. Error bars denote standard deviation (SD). Degree of polymerization (DP).

**Figure 2:**
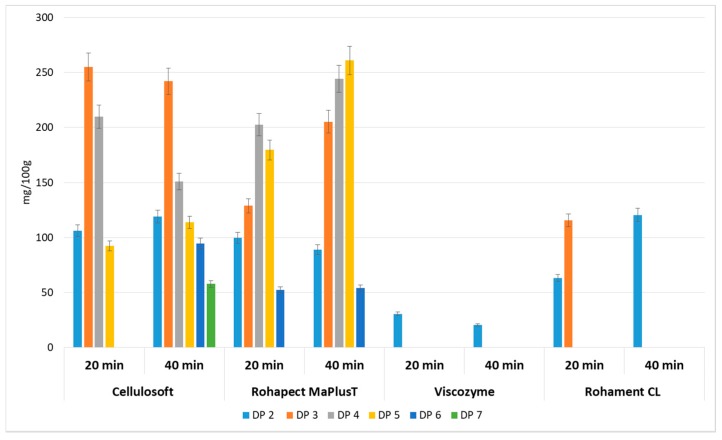
Yields of oligosaccharides with different degrees of polymerization obtained after enzymatic hydrolysis of apple pomace using cellulases and pectinases separately. Data represent mean from two independent experiments repeated three times. Error bars denote SD.

Apple pomace was blended with water in proportions of 1:3 (*w/w*). The pH of the mixture was adjusted to 4.0 using 0.1 M NaOH. Combinations of pectinolytic enzymes (Pectinex Ultra SP-L, Viscozyme, Rohapect Ma Plus T, Rapidase Smart) at a concentration of 75 ppm and cellulolytic enzymes (Cellulosoft 1.5L, Rohament CL) at a concentration of 37 ppm were used. The samples were incubated at 40 °C for different incubation times, as indicated in Figure 1 and Figure 2. The mild conditions of AP hydrolysis was optimized taking into account sensory features of the final product. Avoiding the aroma, which is nonspecific to apples (burned, cooked, caramel aroma) was crucial, since the sensory characteristic is one of the most important features of food additives.

### 2.4. High-Performance Anion-Exchange Chromatography

Chromatographic analysis was performed using a DIONEX ICS-3000 ion chromatograph (Dionex, Chelmsford, MA, USA) with an electrochemical detector PED produced by DIONEX (USA). Oligomers were separated using the Dionex Carbo Pac PA200 (3 × 250 mm) analytical column and a Dionex Carbo Pac PA200 guard column (3 × 50 mm). The separation of oligosaccharides was conducted under the following conditions: Flow rate 0.8 mL/min, column temperature 27 °C, injection volume 25 μL. Gradient separation was used with the following eluents applied as the mobile phase: Eluent 1:200 mM NaOH (solution 50% *v/v* in water NaOH, Sigma-Aldrich^®^, Europe) in 550 mM NaOAc (Sigma-Aldrich^®^, Europe); eluent 2: 250 mM NaOH (solution 50% *v/v* in water NaOH, Sigma-Aldrich^®^, Europe); eluent 3: distilled water (18 MΩ). The following time and composition programs were used (eluents 1, 2, and 3 were expressed as percentage (*v/v*)): From −3 to 0 min: 0, 80, and 20; from 0 to 5 min: 0, 80, and 20; from 5 to 45 min: 45, 55, and 0; from 45 to 90 min: 90, 10, and 0; from 90 to 95 min: 90, 10, and 0; from 95 to 100 min: 0, 80, and 20. Chromatographic analysis was recorded using the Chromeleon^®^ program, version 6.80. Mono-, di- and tri-galacturonic acid (Sigma) as well as higher oligomers DP4-DP10 (obtained by hydrolysis of polygalacturonic acid [17]) were used as standards.

### 2.5. Strains of Lactic Acid Bacteria

The research used 11 strains of *Lactobacillus* sp. bacteria: *Lb. plantarum* ŁOCK 0981, ŁOCK 0982, ŁOCK 0989, ŁOCK 0990, ŁOCK 0991, ŁOCK 0995, ŁOCK 0996; *Lb. brevis* ŁOCK 0983, ŁOCK 0984; *Lb. paracasei* ŁOCK 0985, ŁOCK 0993 and two strains of *Leuconostoc mesenteroides*, ŁOCK 0986 and ŁOCK 0994. All of the strains were acquired from the collection at the Institute of Fermentation Technology and Microbiology (ŁOCK 105), Łódź University of Technology, Poland. The strains were stored with the usage of Cryobanks™ (Copan Diagnostics Inc., 26055 Jefferson Ave, Murrieta, CA 92562, USA) in a temperature of −22 °C. Before experiments, lactic acid strains were activated and passaged twice in the Man, Rogosa and Sharpe broth (MRS; Merck, Darmstadt, Germany) for 24 h in 37 °C.

### 2.6. Pathogens and Fecal Isolates

The following pathogens were used in the adhesion assay: *Escherichia coli* (ATCC 10536), *Salmonella enterica* serovar Typhimurium (ATCC 14028) and *Listeria monocytogenes* (ATCC 19115). These strains were stored in Cryobanks™ (Copan Diagnostics Inc., 26055 Jefferson Ave, Murrieta, CA 92562, USA) in the temperature of −22 °C. Before the experiment, strains were activated and passaged twice in Nutrient broth (Merck, Darmstadt, Germany) for 24 h in 37 °C, without oxygen limitation.

In addition, fecal bacteria (aerobic and anaerobic) were isolated from feces according to the following procedure: A fresh fecal sample was collected in a sterile plastic container, transported at 4 °C under anaerobic conditions, and submitted to analysis within 2–3 h. The sample of feces was supplemented with sterile nutrient broth containing 2% glucose (20%/80%, *v/v*) (Merck, Darmstadt, Germany) and homogenized in a stomacher for 2 min. The homogenized samples were transferred into tubes sealed with special caps (Roth) and incubated at 37 °C for 72 h under anaerobic and aerobic conditions. Medium for anaerobes was additionally supplemented with L-cysteine (0.5 g/L). The tubes for anaerobes isolation were incubated at 37 °C for 72 h with AnaeroGen paper sachets (Oxoid) in anaerobic jar. Before further analysis, the cultures were passaged twice in nutrient broth (Merck, Darmstadt, Germany).

In the adhesion assay, the pathogens were plated on appropriate media: *E. coli* on MacConkey agar (Merck, Darmstadt, Germany), *S.* Typhimurium on SS agar (Merck, Darmstadt, Germany), *L. monocytogenes* on Palcam Agar Base (Oxoid, Basingstoke, UK), fecal bacteria (aerobic and anaerobic) on Plate Count Agar (Merck, Darmstadt, Germany).

### 2.7. Caco-2 Cell Culture

Human colon adenocarcinoma cell line Caco-2 (41st passage) were purchased from Cell Line Service GmbH (Eppelheim, Germany) and were cultured in Roux bottles, as described previously [18]. Briefly, the cells were cultured in high-glucose Dulbecco’s Modified Eagle’s Medium (DMEM, Sigma-Aldrich, St. Louis, MO, USA), supplemented with 10% fetal bovine serum (FBS, Thermo Fisher Scientific, Waltham, MA, USA), 4 mM GlutaMAXTM (Thermo Fisher Scientific, Waltham, MA, USA), 25 mM HEPES buffer (Sigma-Aldrich, St. Louis, MO, USA), and 100 µg/mL streptomycin/100 IU/mL penicillin mixture (Sigma-Aldrich, St. Louis, MO, USA) for 5 days at 37 °C in the atmosphere of 5% CO_2_. Every 2–3 days, cells were washed with phosphate buffer saline (PBS, pH 7.2, Sigma-Aldrich, St. Louis, MO, USA) and medium was renewed. Confluent cells were detached with TrypLETM Express (Thermo Fisher Scientific, Waltham, MA, USA) for 5 min, according to manufacturer’s instructions. The cell suspension was centrifuged (182× *g*, 5 min), decanted, and the pellet was re-suspended in fresh DMEM. After determination of cell count by hemocytometer and cell viability by trypan blue exclusion, the cells were ready to use [18].

### 2.8. Adhesion Assay

For the adhesion assays, Caco-2 cells were placed in a 24-well plate in the presence of AP pomace hydrolysates (pH 7.0 ± 0.1) at appropriate concentrations (2.5 × 10^5^ cells/well) and left overnight. POS concentration in P1, P2, and P3 hydrolysate was equal to 5.9 g/100 g d.w. All the bacteria were cultured over 24 h at 37 °C. The cultures of *Lactobacillus* sp. and *Leuconostoc* sp. bacteria were diluted in sterile MRS to bring the number of bacteria to 3.5 × 10^9^ CFU/mL for *Lactobacillus* sp. and 8.0 × 10^8^ CFU/mL for *Leuconostoc* sp. (initial numbers), as estimated spectrophotometrically from the standard curves. The initial numbers of pathogens and fecal aerobic and anaerobic bacteria (10^9^ CFU/mL) were determined using a DEN-1 McFarland densitometer (Biosan, Riga, Latvia). Next, the bacteria were centrifuged and the supernatants removed. The pellets were washed with sterile phosphate buffer saline (PBS, Sigma, pH 7.2) to remove the residual substrate, and centrifuged again. Apple pomace hydrolysates were added to the bacterial pellets, while DMEM medium without supplements was used for the control sample. The DMEM medium was removed from the Caco-2 cells in the 24-well plate and 1 mL of the prepared suspensions was added. Each of the samples was prepared in three replicates. The plate was incubated for 2 h at 37 °C in 5% CO_2_ with humidity >95%. After 2 h, non-adhered microorganisms were removed by washing with PBS. To detach the Caco-2 cells with the adhered bacteria, 1% trypsin was added to each well with incubation for 10 min at 37 °C. After this time, the detachment of the cells was observed under an inverted microscope. Cells with adhered bacteria (in PBS) were transferred into sterile Eppendorf tubes, then they were centrifuged and the supernatant was decanted. The resulting pellet was resuspended in 0.1% Triton X-100 and incubated for 5 min at room temperature to lyse Caco-2 cells. The adhered bacteria were counted by the plate method using suitable agar media and incubated at 37 °C for 24–48 h.

In the adhesion test for the lactic acid bacteria, the stimulation rate (%) was calculated as follows:

A = (adhesion of tested sample × 100/adherence of control) − 100.

This is the percentage of the increased adhesion of lactic acid bacteria in the presence of AP.

For the pathogens, the inhibition rate (%) was calculated as follows:

I = 100 − (adherence of tested sample × 100/adherence of control).

This is the percentage of the reduction in adhesion by pathogens in the presence of AP.

### 2.9. Microscopic Observations

For microscopic visualization, the following procedure was carried out using a Lab-TekTM microplate 8-well (Nunc, Thermo Fisher Scientific, Waltham, MA, USA). After incubation of the tested strains for 2 h with AP hydrolysates and Caco-2 cells, non-adhered bacteria were removed. The wells were washed with PBS (pH 7.2) and fixed with 70% methanol for 15 min at ambient temperature. They were then stained with 0.5% crystal violet (Sigma-Aldrich, St. Louis, MO, USA). After that, the wells were washed with 70% ethanol and dried overnight. The samples were observed at ×1000 magnification under a microscope (Nikon, Tokyo, Japan) connected to a camera (Nikon Digital Sight DS-U3, Nikon, Tokyo, Japan) using imaging software (NIS-elements BR 3.0, Nikon, Tokyo, Japan).

In the adhesion assay—during 2-h incubation there was no influence of AP pomace on morphology or monolayer of Caco-2 cells (it was controlled in the inverted microscope). In some cells after 2-h incubation some vacuolization appeared, but the time was too short to influence the morphology or monolayer of the cells in the concentration of AP tested.

### 2.10. In Vitro Fermentability of Pectin-Derived Oligosaccharides (POS)

The prebiotic potential of high- and low-order oligosaccharides was assessed in terms of its ability to support the growth of microbiota from the human gastrointestinal tract. Feces were obtained from four healthy volunteers (non-smoking, without a history of gastrointestinal tract disorders, on basal diet, who had not received antibiotics, probiotics, prebiotics, and any drugs during at least three months before the study) of different ages: 10, 23, 47, and 69 years old. Four individuals with such a wide age distribution were recruited due to the fact that the number and diversity of gut microflora varies with age. To obtain a starting inoculum, 1 g of mixed human feces was introduced into a test tube with 20 mL of sterile 0.85% saline solution. After vortexing, 0.5 mL of the inoculum was introduced into 60 mL of a sterile POS preparation—P1 or P2—and incubated at 37 °C for 21 days (pH = 7 ± 0.1). POS concentration in P1 and P2 hydrolysate was equal to 5.9 g/100 g d.w. The numbers of *Lactobacillus*, *Bifidobacterium*, *Clostridium*, *Bacteroides*, *Enterococcus*, and Enterobacteriaceae, as well as the total numbers of anaerobic and aerobic bacteria, were determined by plating using selective microbial media (Table 1).

### 2.11. Short-Chain Fatty Acid (SCFA) and Lactic Acid Analysis

High-performance liquid chromatography (HPLC) analysis was carried out using a Thermo Separation Products apparatus (Thermo Scientific, Waltham, MA, USA) equipped with RI Plus and PDA detectors and an Aminex HPX 87H + column (300 × 7.8 mm id) (Bio-Rad, Hercules, CA, USA). The flow rate was 0.6 mL/min, the temperature was 60 °C, and 0.005 M sulfuric acid was used as the phase. Identification and quantification of compounds was carried out based on standard compounds: formic acid, acetic acid, propionic acid, and lactic acid (Sigma) [19].

### 2.12. Statistical Analysis

The data were analyzed using two-way analysis of variance (ANOVA). The differences between samples with normal distributions were evaluated using a Student’s *t*-test. Both the Student’s *t*-test and ANOVA were performed using OriginPro 6.1 software (OriginLab Corporation, Northampton, MA, USA). Significant differences were accepted at *p* < 0.05). The results are presented as the mean ± standard deviation (SD). Principal Component Analysis (PCA) with Varimax rotation was performed using the Statistica 10 program. A 9 × 19 matrix was constructed for the obtained oligosaccharides and different hydrolysis parameters. All experiments were performed in three replicates (only for adhesion assay for pathogens—there were four repeats).

## 3. Results

This study was conducted to identify the composition and the biological activity of pectin-derived oligosaccharides generated from mild acid or enzymatic hydrolysis of apple pomace. The effect of the polymerization of the structural units of POS contained in the AP hydrolysate on the growth and metabolism of microbiota from the human gastrointestinal tract and the adhesion of lactic acid bacteria or pathogens to human gut epithelial cells was investigated in vitro.

### 3.1. Enzymatic Hydrolysis of Apple Pomace

Enzymatic hydrolysis of AP was performed using a mixture of different commercial preparations of cellulase (Cellulosoft 1.5L or Rohament Cl) and pectinase (Viscozyme, Rapidase Smart, Pectinex Ultra SP-L or Rohapect Ma Plus T). The qualitative and quantitative composition of the generated oligosaccharides depended on the enzyme specificity and the period of enzymatic reaction. Enzymes are very effective tools for modifying the structure and functionality of pectin. Even after a seven min reaction time, oligosaccharides with degrees of polymerization ranging between DP2–DP6 and DP2–DP9 were generated, depending on the cellulolytic and pectic enzyme combination. This indicates that the application of enzymes is a feasible method for POS generation. However, the best effects were noted after 20 min of pomace hydrolysis using a mixture of Cellulosoft and Viscozyme, as well as for Rohament CL and Rohapect Ma Plus T, which gave the highest yields of total oligosaccharides, at 5.56 and 5.94 g/100 g d.w., respectively. These concentrations were 3.4- and 3.6-fold higher than the equivalent probe treated with Cellulosoft and Pectinex Ultra SP-L polygalacturonase. With extended enzymatic hydrolysis, a change in the pattern of oligosaccharides was observed. Higher oligomers were hydrolyzed to molecules of lower molecular weight (Figure 1).

Cellulases (Cellulosoft and Rohament CL) and pectinases (Rohapect Ma Plus T and Viscozyme) were applied to AP separately to study their nondependent activities. These treatments resulted in very low amounts of oligosaccharides, ranging from 0.02 to 0.77 g/100 g d.w. (Figure 2). It can be concluded that the application of cellulase in a simultaneous process with pectinase enhances the production of oligosaccharides. It may be assumed that cellulolytic enzymes release pectins from the AP and thus, provide a better environment for pectinase activity.

### 3.2. Mild Acid Hydrolysis Followed by Enzymatic Hydrolysis of Apple Pomace

We used a two-step process for the production of oligosaccharides, involving mild acid hydrolysis of AP followed by enzymatic hydrolysis using three different commercial pectinase preparations: Rohapect MaPlus T (AB Enzymes), Viscozyme (Novozymes), and Pectinex Ultra AFP (Novozymes). Mild acid hydrolysis was used as a pretreatment method, instead of cellulase, to release pectin molecules bound to the AP tissue. On an industrial scale, pectins are extracted from plant cells using a strong mineral acid solution with less than pH 1–3, at temperatures in the range of 60–100 °C, over at least 3 h [20,21]. During acid extraction, the pectin structure undergoes often far-reaching modifications. Many of the branched structures of ramnogalacturonate I or ramnogalacturonate II which are formed by neutral sugars (e.g., arabinans, galactane, xyloglucane) are destroyed. They are more sensitive to acid hydrolysis than bonds combining ramnose with GA in the primary skeleton of RG I or the bonds between GA in HG and RG II. However, the polygalacturonate chain remains stable under such conditions. Commercial pectin preparations are mostly homogalakturonian. Highly degraded RG I and RG II form only a small percentage of the product, and the combined arabians and galactans comprise 0.5%–6.3% and 3.1%–6.2%, respectively [22,23].

**Figure 3:**
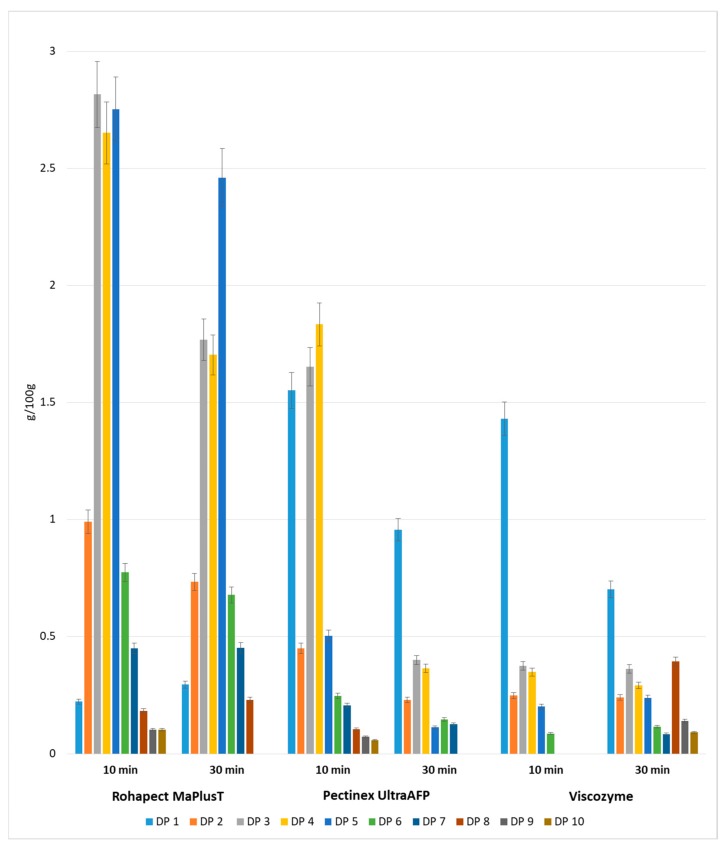
Yields of oligosaccharides with different degrees of polymerization obtained after mild acid and enzymatic hydrolysis of apple pomace. Data represent mean from two independent experiments repeated three times. Error bars denote SD.

In contrast to the pectin extraction method used in this study, the duration of mild acid hydrolysis was optimized for 20 min. For incubation periods longer than 20 min, the total amount of POS dropped dramatically. The DP distributions after mixed hydrolysis are shown in Figure 3. Chromatographic analysis confirmed the presence of a mixture of oligosaccharides with different degrees of polymerization in the range of DP1–DP10 in all the tested samples. Acid hydrolysis followed by Rohapect Ma Plus T was the best method for producing large amounts of oligosaccharides, especially for short periods of enzymatic hydrolysis (10 min), at 10.81 g/100 g d.w. Moreover, applying acid hydrolysis followed by Rohapect Ma Plus T resulted in very low release of galacturonic acid (0.22 g/100 g d.w.). Its concentration after 10 min of incubation time was between 6.5-fold and 7-fold lower, in comparison to the samples with Viscozyme and Pectinex AFP, respectively. In the sample of AP treated with Viscozyme, no oligosaccharides with a DP higher than 6 were noticed after 10 min of enzymatic hydrolysis. The lowest concentration of total oligosaccharides was also noted, at 1.25 g/100 g d.w.

### 3.3. Comparison of Applied Hydrolytic Methods

Figure 2 shows the quantitative and qualitative composition of two representative samples, in which the highest yields of oligosaccharides were obtained by different hydrolytic methods: mild acid hydrolysis followed by Rohapect Ma Plus T, or enzymatic hydrolysis using a mixture of Rohament CL and Rohapect Ma Plus T. It was found that mild acid hydrolysis followed by pectinolysis was more favorable for the production oligosaccharides from AP, resulting in a 1.8-fold higher overall yield of oligosaccharides. However, despite the lower total yield of POS, the AP sample treated only by enzymatic hydrolysis contained higher amounts of oligosaccharides with higher-order DPs (7–9 DP). The oligosaccharide yields DP7 > DP8 > DP9 were 0.6 > 0.4 > 0.3 g/100 g d.w. for enzymatic hydrolysis and 0.4 > 0.2 > 0.1 g/100 g d.w. for acid and enzymatic hydrolysis. The total number of DPs at 7–9 after enzymatic hydrolysis was 1.7-fold higher than in the hydrolysate obtained using combined hydrolysis. Taking into account the high levels of higher-order DPs (7–9 DP), the hydrolysate obtained using the pure enzymatic method appeared to exert valuable biological activity.

**Figure 4:**
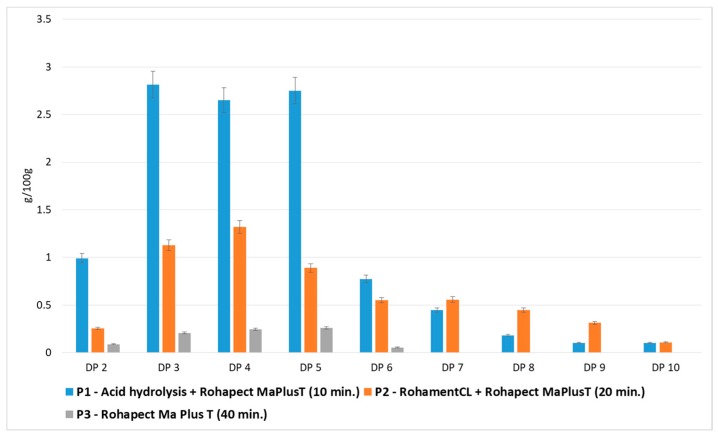
Effects of enzymatic and mild acid hydrolysis of apple pomace in comparison to enzymatic hydrolysis in terms of pectin-derived oligosaccharides (POS) production. Data represent mean from two independent experiments repeated three times. Error bars denote SD.

Therefore, the samples: P1—after mild acid hydrolysis followed by Rohapect Ma Plus T, P2—after enzymatic hydrolysis using a mixture of Rohament CL and Rohapect Ma Plus T, as well as the P3 sample containing only lower-order DPs (2-6 DP) prepared using Rohapect Ma Plus T (Figure 4), were submitted to analysis to determine their bifidogenic and antiadhesive properties.

### 3.4. Relationship Between the Type of POS Obtained and the Hydrolytic Method

A PCA test was performed to reveal the relationship between the type of POS released and the type of processing method used. By analyzing the percentages of variance, it was observed that 85.79% of the total variance was captured by only two components. The principal component (PC1) contributed 55.70% and the secondary component (PC2) accounted for 30.09% of the variance. The PCA1 group included DP_PCA1_ = [2, 3, 4, 5, 6, 7], while DP_PCA2_ = [8, 9, 10]. The DP7 dimension was classified to PCA1 on the basis of a slightly higher factor coefficient. However, the DP7 dimension may also be considered as belonging to PCA2 (Table 2).

**Figure 5:**
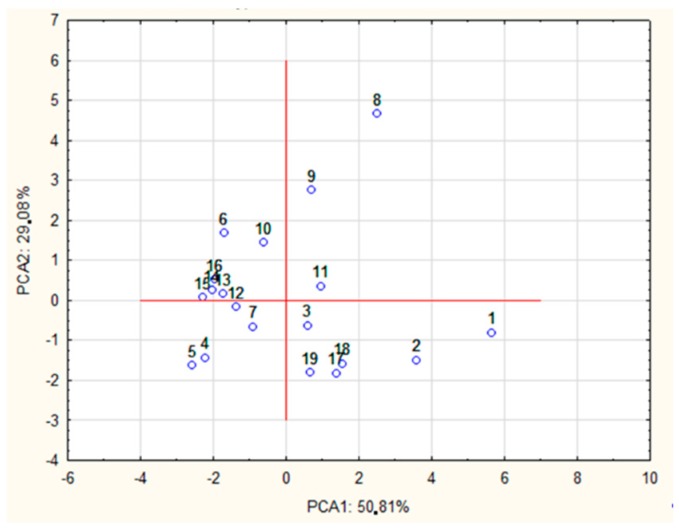
PCA of the olidosaccharides obtained by different hydrolytic treatments, where 1—AH + Rohapect MaPlusT (10 min), 2—AH + Rohapect MaPlusT (30 min), 3—AH + Pectinex UltraAFP (10 min), 4—AH + Pectinex UltraAFP (30 min), 5—AH + Viscozyme (10 min), 6—AH + Viscozyme (30 min), 7—Cellulosoft + Viscozyme (7 min), 8—Cellulosoft + Viscozyme (20 min), 9—Cellulosoft + Viscozyme (40 min), 10—Cellulosoft + Viscozyme (60 min), 11—Cellulosoft + Viscozyme (80 min), 12—Cellulosoft + Pectinex UltraSPL (7 min), 13—Cellulosoft + Pectinex UltraSPL (20 min), 14—Cellulosoft + Pectinex UltraSPL (40 min), 15—Cellulosoft + Pectinex UltraSPL (60 min), 16—Cellulosoft + Pectinex UltraSPL (80 min), 17—Rohament Cl + Rohapect MaPlusT (10 min), 18—Rohament Cl + Rohapect MaPlusT (20 min), 19—Rohament Cl + Rohapect MaPlusT (30 min). * Different hydrolytic treatments were described as: Enzyme type (hydrolysis time); AH—acid hydrolysis.

In the final part of the study, case classification was performed on the basis of the correlation for each PCA factor. PCA2 may be explained by pure enzymatic processing using a mixture of Cellulosoft and Viscozyme as well as Rohament CL and Rohapect Ma Plus T. As can be seen in Figure 5, the samples were divided into several clusters according to the method of AP processing used. Regardless of the type of acid processing used, all the samples treated with Rohapect Ma Plus T were placed in the fourth quarter of the coordinate system.

### 3.5. Adhesion of Lactic Acid Bacteria in the Presence of Apple Pomace Hydrolysates

**Figure 6:**
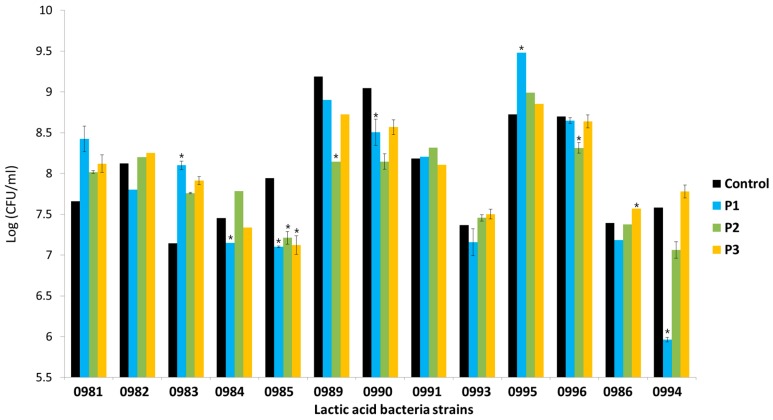
Number of adhered bacteria in the control sample and in the presence of apple pomace hydrolysates: P1 (oligosaccharides with DP 1–10), P2 (oligosaccharides with DP of 1–10 but twice the content of oligosaccharides with DP 7–10 than in P1), and P3 (oligosaccharides with DP 1–6). Data represent mean from three repeats in one experiment. Error bars denote SD. * Results significantly different from unexposed control, ANOVA (*p* < 0.05).

All the tested strains showed strong adhesion to Caco-2 cells. The number of adhered bacteria in the control sample and in the samples incubated with AP hydrolysate depended on the strain (Figure 6). Among the control samples, the highest adhesion of bacteria to Caco-2 cells was observed for *Lb. plantarum* 0989 and 0990 (above 1 × 10^9^ CFU/mL of adhered bacteria). The least adhesive was *Lb. brevis* 0983 (1.4 × 10^7^ CFU/mL of adhered bacteria). These abilities also depended on the type of prebiotic preparation. The presence of P1 preparation, containing oligosaccharides with DP 2–10, reduced the number of adhered bacteria by 1 logarithmic unit in most cases (9 out of 13 strains). The presence of preparation P2 appeared to increase the number of adhered *Lactobacillus* sp. bacteria (7 out of 11 strains). Only preparation P3, which contained oligosaccharides with DP 1–6, caused an increase in the number of adhered *Leuconostoc* sp. bacteria.

**Figure 7:**
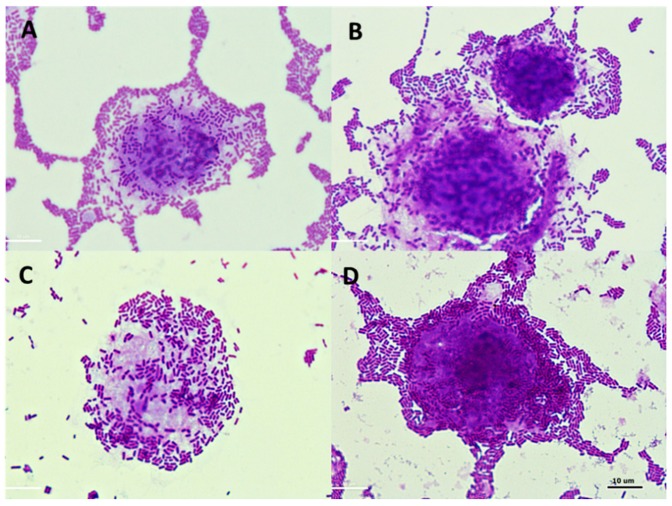
Microphotographs (magnification × 1000) showing adhesion of *Lactobacillus* sp. to Caco-2 cells in the presence of and without apple pomace hydrolysates: (**A**) *Lb. plantarum* 0991, (**B**) *Lb. plantarum* 0991 + preparation P1, (**C**) *Lb. plantarum* 0989, (**D**) *Lb. plantarum* 0989 + preparation P3.

The impact of the POS preparations on the adhesion of the tested strains to Caco-2 cells was assessed in terms of the stimulation rate ((adherence in the presence of POS × 100/adherence in control sample) − 100). The use of different AP hydrolysates affected the adhesion of the tested lactic acid bacteria (Table 3). These changes depended to a great extent on the strain. Among the *Lactobacillus* sp. strains, the highest increase in adhesion to Caco-2 cells after 2 h of incubation with POS was shown by *Lb. plantarum* 0981, 0995, and *Lb. brevis* 0983. This increase was observed for all the hydrolysates. The highest decrease in adhesion was observed for *Lb. paracasei* 0985, *Lb. plantarum* 0989, 0990, and 0996. Preparation P2 stimulated the adhesion of the highest number of strains, at 7 out of 13 Hydrolysate P3 stimulated 5 of the 11 tested strains. Preparation P1 stimulated 3 of the 13 strains. For *Leuconostoc* sp., stimulation of adhesion to Caco-2 cells was observed only in the presence of preparation P3 (increase in adhesion approx. 50%–60%). The other hydrolysates reduced the number of adhered bacteria. The largest decrease was observed in the presence of preparation P1 (21.3% lower adhesion as compared with the control for *Leu. mesenteroides* 0994). The adhesion of the tested lactobacilli to Caco-2 cells in the presence of AP preparations (P1, P2, and P3) and in the control (medium + strain) is presented in Figure 7.

### 3.6. Adhesion of Pathogens and Fecal Bacteria in the Presence of Apple Pomace Hydrolysates

**Figure 8:**
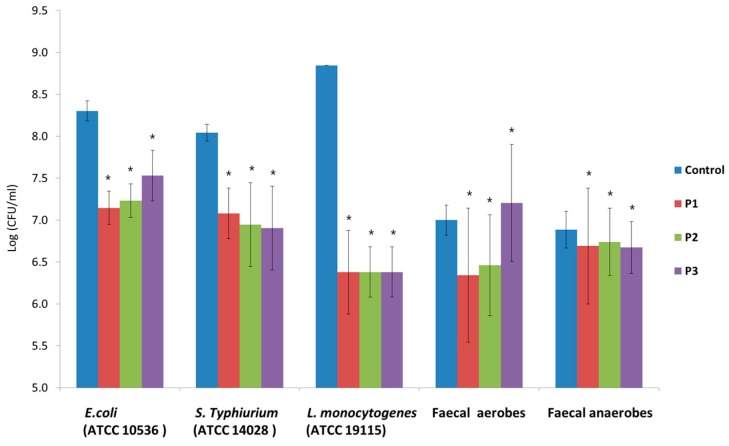
Number of pathogens and fecal isolates adhered to Caco-2 cells in the presence of and without apple pomace (P1, P2, and P3) ± SD. Data represent mean from four repeats in one experiment. Error bars denote SD. * Results significantly different from the unexposed control, ANOVA (*p* < 0.05).

The applied pathogen strains adhered in large amounts to the Caco-2 cells. The most adhesive was *L. monocytogenes* (ATCC 19115), with 7.0 × 10^8^ CFU/mL of adhered bacteria, and the least adhesive was *S.* Typhimurium (ATCC 14028), with 1.1 × 10^8^ CFU/mL of adhered bacteria. The tested fecal aerobes and anaerobes adhered to the Caco-2 cells at similar levels (approx. 1.0 × 107 CFU/mL of adhered bacteria). In the presence of the applied AP hydrolysates, a reduction was observed in the number of adhered bacteria in the cases of all the tested pathogens (Figure 8). These changes were at a similar level in the presence of all three preparations. The largest reduction in the number of adhered pathogens after incubation with AP hydrolysates was observed for *L. monocytogenes* (ATCC 19115) (more than 2 log units). For aerobic fecal bacteria, reductions in the numbers of microorganisms were observed in the presence of P1 and P2 preparations. After 2 h of incubation in the presence of the hydrolysates, there was a slight reduction in the number of anaerobic fecal bacteria adhered to Caco-2 cells in comparison to the control sample.

The impact of the AP hydrolysates on the adhesion of pathogens and fecal isolates was evaluated based on the stimulation/inhibition rate (%), which was calculated as follows: (adherence of tested sample × 100/adherence of control)—100%. Of the tested pathogens, the greatest reduction in adhesion was observed for *L. monocytogenes* (ATCC 19115) (approximately 28%). In the cases of *E. coli* (ATCC 10536) and *S.* Typhimurium (ATCC 14028), this decrease was 9.3%–14.2%, depending on the hydrolysate. The weakest inhibition of adhesion in the presence of AP was observed for fecal anaerobic bacteria (approximately 2.1%–3.1%). It was further observed that in the presence of preparation P3 there was an increase in the adhesion of aerobic bacteria (Table 4).

**Figure 9:**
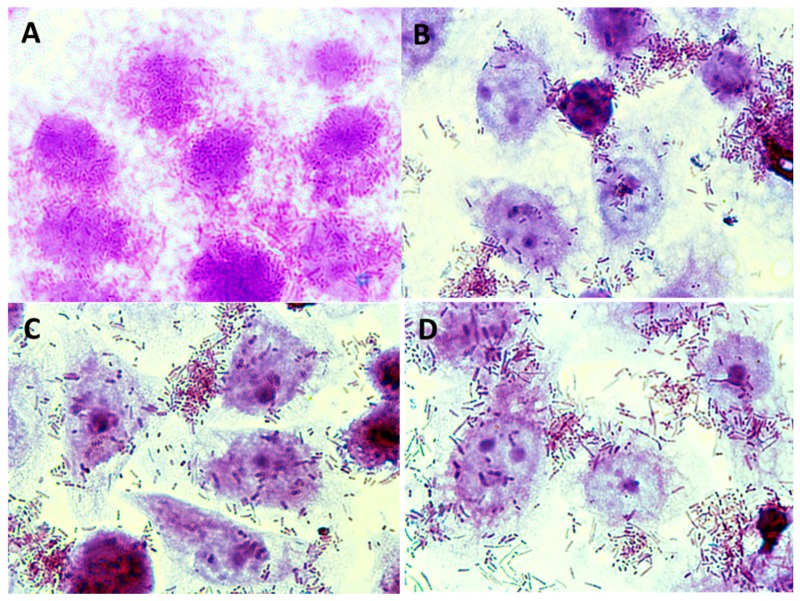
Microphotographs (magnification × 1000) showing adherence of *E. coli* ATCC 10536 to Caco-2 cells in the presence and without apple pomace hydrolysates: (**A**) *E. coli* ATCC 10536, (**B**) *E. coli* ATCC 10536 + P1 preparation, (**C**) *E. coli* ATCC 10536 + P2 preparation, (**D**) *E. coli* ATCC 10536 + P3 preparation. Stained with 0.5% crystal violet.

**Figure 10:**
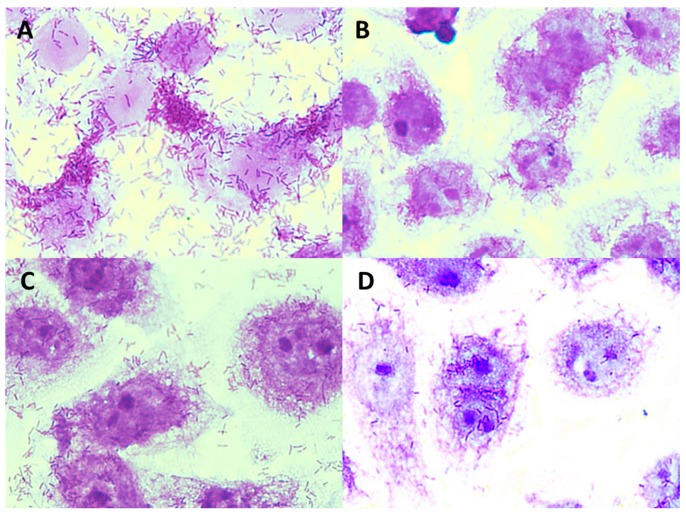
Microphotographs (magnification × 1000) showing adhesion of *L. monocytogenes* ATCC 19115 to Caco-2 cells in the presence and without apple pomace hydrolysates: (**A**) *L. monocytogenes* ATCC 19115, (**B**) *L. monocytogenes* ATCC 19115 + P1 preparation, (**C**) *L. monocytogenes* ATCC 19115 + P2 preparation, (**D**) *L. monocytogenes* ATCC 19115 + P3 preparation. Stained with 0.5% crystal violet.

The adhesion of the tested pathogens to Caco-2 cells in the presence of AP preparations (P1, P2, and P3) and in the control (medium + strain) is presented in Figure 9 and Figure 10.

### 3.7. Effect of POS Composition on the Growth and Metabolism of Microbiota from the Human Gastrointestinal Tract In Vitro

**Figure 11:**
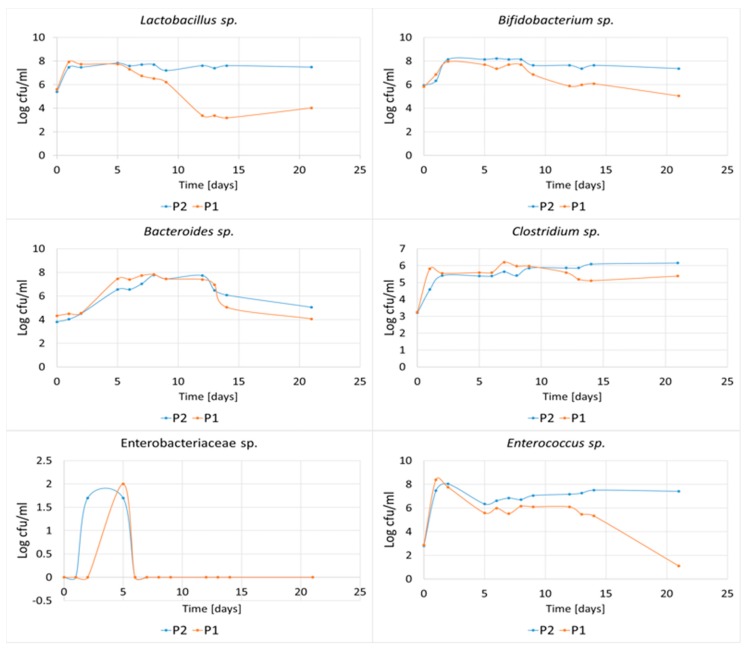
Dynamics of growth of *Lactobacillus, Bifidobacterium, Clostridium, Bacteroides, Enterococcus* and *Enterobacteriaceae* bacteria during in vitro fermentations in P1 and P2 hydrolysates. Data represent mean from three repeats in one experiment. Error bars denote SD.

In vitro fermentations were carried out to confirm the effect of the ratio of higher-order oligosaccharides contained in the P1 and P2 hydrolysates on the growth of mixed bacteria populations from the human colon (Figure 11). Intense growth of *Lactobacillus* and *Bifidobacterium* was observed within the first two days of incubation (the number of bacteria increased on average by two logarithmic units, for both the P1 and P2 media). However, in the case of the P1 hydrolysate, there was a notable decrease in the levels of these bacteria after 5 days (*Lactobacillus*) and 8 days (*Bifidobacterium*) of incubation. After 21 days, the population of *Lactobacillus* decreased by 48.0% and the population of *Bifidobacterium* fell by 34.4%. In contrast, these bacteria were able to maintain constant population in the P2 hydrolysate over 21 days, indicating that the higher-order oligosaccharides contained in this medium had excellent prebiotic properties.

Higher number of *Bacteroides* after 21 days of fermentation was also observed in the P2 hydrolysate containing larger amounts of higher-order POS. Some *Bacteroides* sp. are known for their ability to degrade plant-derived polysaccharides in the gut and produce intermediates for energy. These bacteria have also been considered as candidates for new probiotics [24]. Bacteria of the *Enterobacteriaceae* sp. were inhibited after the fifth day of incubation and were not able to grow in the tested hydrolysates after six days. *Enterococcus* bacteria also exhibited decreasing growth rates after 12 days of fermentation of POS contained in P1 hydrolysate. *Clostridium* bacteria showed constant CFU number/mL during the incubation period in both the P1 and P2 hydrolysates.

**Figure 12:**
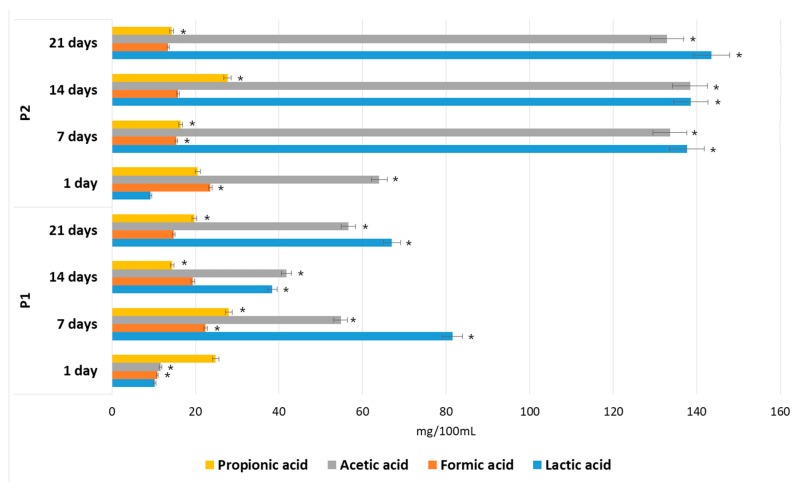
Short-chain fatty acids (SCFA) and lactic acid concentrations during in vitro fermentations in P1 and P2 media. Data represent mean from three repeats in one experiment. Error bars denote SD. * Significant differences between P1 and P2 hydrolysate for each fermentation period, ANOVA (*p* < 0.05).

Figure 12 shows the effects of the P1 and P2 hydrolysates on SCFA accumulation during in vitro fermentation performed by fecal microorganisms. The main metabolites in the cultures with both apple pomace extracts, P1 and P2, were lactic acid and acetic acid. *Lactobacillus* and *Bifidobacterium* are considered to be the main producers of lactic acid in the human gut, whereas acetic acid may be generated by many anaerobic bacteria. The 14.8-fold increase in the concentration of lactic acid and the 2.1-fold increase in acetic acid during the first 7 days of fermentation, followed by the constant levels of these compounds during the rest of the period (days 7–21), correlate well with the increased number of *Lactobacillus, Bifidobacterium* and total anaerobes in the P2 medium. The reduced amounts of these metabolites in the P1 media (by 59.2% and 41.0% after 7 days and by 46.2% and 42.6% after 21 days of incubation) are due to the lower and decreasing populations of these bacteria in those media, compared with the P2 media.

Propionic acid (19.7 mg/100 mL in P1; 14.31 mg/100 mL in P2) and formic acid (14.8 mg/100 mL in P1; 13.4 mg/100 mL in P2) were also found. However, the quantities were low compared with the levels of total of SCFA and lactic acid found in hydrolysates P1 (158.1 mg/100 mL) and P2 (304.1 mg/100 mL). High levels of acid production due to POS fermentation is considered desirable, since it has been proven that low pH values and SCFA accumulation inhibit the growth of pH-sensitive gut pathogens, such as *E. coli* and *Salmonella* [25]. In our study, *Enterobacteriaceae* sp. were inhibited after the fifth day of incubation and were unable to grow in the tested hydrolysates after six days. This correlates well with the accumulation of SCFA and lactic acid in the fermentation media.

## 4. Discussion

### 4.1. The Effect of the Polymerization of POS Contained in the AP Hydrolysate on the Growth and Metabolism of Microbiota from the Human Gastrointestinal Tract

Changes in microbial population dynamics and metabolic activity related to non-digestible dietary fiber content have been well documented [26,27,28,29]. Gut microbiome modulation is an important tool, providing health benefits to the host mainly due to the increased generation of SCFA in the large intestine. Acetate, propionate and butyrate are the main products of the saccharolytic fermentation of non-digestible carbohydrates, which have been shown to exert multiple beneficial effects on the mammalian metabolism. Approximately 95% of the SCFA originating from microbiota fermentation are absorbed into the bloodstream, generating systemic actions closely related to anti-inflammatory, anti-tumor, anti-obesity, dyslipidemic, anti-diabetic and antimicrobial effects [30,31,32,33].

Differences in the metabolism of oligosaccharides with origins other than POS (inulin-type fructans and galacto-oligosaccharides) have been reported by Wilson et al. (2017) [34]. Both Bifidobacteria and Lactobacillus genera have been shown to selectively ferment prebiotic fiber, based on the enzyme characteristics of the bacterial population. Moreover, Gullon et al. (2011) observed that pure Bifidobacterium strains and Bacteroides vulgatus cultures consumed the various fractions in a mixture of AP oligosaccharides (gluco-, galacto-, xylo- and arabinooligosaccharides) at different rates, as well as the SCFA patterns. Moon et al. (2015) [35] showed the different prebiotic effects of linear arabino-oligosaccharides (LAOS) and debranched (linear) sugar beet arabinan (LAR).

In our study, it was observed that a higher ratio of higher-order oligosaccharides (DP 7–10) caused an increase in the prebiotic effect of the AP hydrolysate, inducing changes both in the counts of intestinal bacteria groups and in the amount and nature of SCFA produced. The various microorganisms in the fecal samples enable the complete fermentation of carbohydrates in the gut. In the mixed cultures of fecal microorganisms studied in vitro on apple pomace hydrolysates, the growth of bacteria of the genus *Lactobacillus* and *Bifidobacterium* was more stimulated by structurally higher oligosaccharide molecules. However, the modification of other microbial groups (e.g., *Clostridium*, *Bacteroides*, *Enterococcus*, and *Enterobacteriaceae* bacteria) should be also considered, due their ability to utilize acetate and lactate generated during oligosaccharide consumption by probiotic microorganism groups, or to utilize sugars generated as products of oligosaccharide breakdown during the cross-feeding mechanism [36].

### 4.2. The Effect of the Polymerization of POS Contained in the AP Hydrolysate on the Adhesion of Lactic Acid Bacteria (LAB) or Pathogens to Human Gut Epithelial Cells

Adhesion is an important process for the survival and proliferation of probiotic bacteria in the digestive tract. It is believed that mere passage through the intestinal tract is insufficient to cause health effects [37]. Since the adhesion of bacteria to the intestinal epithelium affects their residence time in the gastrointestinal tract, this ability is considered one of the most important criteria for the selection of probiotic strains [38]. In vivo studies on the adhesion of microorganisms are difficult to perform, and therefore in vitro models are often used [39]. To select probiotic strains, frequently used models include cell lines of human origin such as the small intestine. These can provide a great deal of information on the interactions between probiotics and host cells [38]. The beneficial effects of probiotics may be strengthened by using prebiotics. These stimulate the growth of positive micro-organisms which reside in the digestive system [40]. Apple pomace may be a potential source of POS prebiotics.

In the research presented here, it was found that all the applied hydrolysates of AP stimulated the adhesion of 3 of the 13 tested strains of lactic acid bacteria. These were *Lb. plantarum* 0981, 0995, and *Lb. brevis* 0983. In the case of the other strains, changes in bacterial adhesion to the intestinal cells depended on the AP preparation. Preparations P2 and P3 stimulated the adhesion of the greatest number of tested strains (7 of the 13 strains). However, the level of stimulation was significantly different depending on the strain (from 0.3% to 13.4%). The highest increases in the number of adhered *Lactobacillus* sp. bacteria were observed for *Lb. plantarum* 0981 and *Lb. brevis* 0983 in the presence of preparation P1 (10.0% and 13.4%, respectively). Only preparation P3 stimulated the adhesion of *Leu. mestenteroides*. Although the adhesion of microorganisms can be affected by many factors, there is little information on the effects of prebiotics in the process of adhesion by probiotic strains. A few studies have focused on the effects of prebiotics or single saccharides on the adhesion of probiotics. Koh et al. (2013) [41] observed that the addition of tagatose stimulated the adhesion of *Lb. rhamnosus* GG and *Lb. casei* 01 to HT-29 cells by approximately 7%. Kavanaugh et al. (2013) [42] studied the effect of various prebiotic oligosaccharides on the adhesion of a strain with confirmed probiotic properties (*Bifidobacterium longum* subsp. *infantis* ATCC 15697) to Caco-2 and HT-29. A combination of 3′- and 6′-sialyllactose and the use of 6′-sialyllactose separately increased the adhesion of the tested strain. The use of a combination of 3′-sialyllactose and commercial prebiotic Orafti P95 did not increase the adhesion of the strain. Incubation with lactose and oligofructose did not result in a significant increase in the adhesion of the test strain to Caco-2 cells.

Kadlec and Jakubec (2014) [37] suggest that interactions between pre- and pro-biotics vary and depend strongly on the strain. They report that prebiotics and saccharides caused a decrease in the adhesion of lactic acid bacteria to Caco-2 and HT-29. However, in some combinations there was an increase in adhesion after incubation with prebiotics. One was *Lb. rhamnosus* CCDM 150, which after incubation with P95 or the prebiotic Vivinal, showed increased adhesion. This increase was probably not due to the presence of simple sugars, which are often present in residual amounts in commercial prebiotics, since the tested strains also showed reduced adhesion after incubation with lactose, glucose and galactose. Similar results were obtained by Krausova et al. (2016) [43], who studied the effects of three commercial prebiotic preparations on the adhesion of five *Lactobacillus* strains to intestinal mucus. These authors observed that in most cases the addition of prebiotic resulted in the reduction of adhesion. The only exception was the strain *Lb. gasseri* PHM-7E1, to which the addition of Orafti prebiotics GR and Orafti P95 resulted in increased adhesion. However, the addition of a mixture of the prebiotics caused a reduction in adhesion to mucin. In addition, the addition of glucose appeared to reduce the adhesion of lactic acid bacteria.

The available data suggest that in particular strains the process of adhesion to the intestinal epithelium proceeds according to different mechanisms. These mechanisms have, in general, yet to be described. It is still not properly understood why two prebiotics may have different effects on the adhesion of the same strain [37]. Russo et al. (2012) [44] showed that *Lb. plantarum* WCFS1 after incubation in the presence of β-D-glucan showed a five-fold increase in adhesion to Caco-2 cells. Tamminen et al. (2013) [45] studied the ability of *Lactobacillus* bacteria to ferment carrot juice and its effect on the adhesion of the tested strains to intestinal mucus. They found that after fermentation the juice strains showed reduced adhesion (e.g., in the case of *Lb. plantarum* 115 adhesion declined from 2.7% to 1.0%). Based on their results, the authors suggest that the adhesion ability of bacteria may be changed after culturing in different media. Ouwehand et al. have shown that the adhesion of bacteria is reduced in low pH environments. Therefore, attention should be given to the impact of the food media, because it may affect the functionality of probiotics [46].

The adhesion of pathogenic bacteria is an important factor in causing infections [47]. The process is facilitated by having pili and fimbriae, which are present in many Gram-negative bacteria, especially species of the *Enterobacteriaceae* family. These appendages allow adhesion to the intestinal epithelium in both humans and animals [48]. It has been found that carbohydrates can inhibit microbial growth in the digestive tract by preventing their adhesion to the intestinal epithelium [49]. This compound resembles the saccharides present on the surface of epithelial cells, which are part of glycoproteins. These are the places to which pathogens adhere. Thus, the carbohydrates may act as a decoy receptor that decreases the binding of pathogens to the intestinal mucus [50]. The use of anti-adhesive compounds of natural origin offer an important alternative to antibiotic therapy. Examples include the compounds in cranberry juice [51] or oligosaccharides in human milk [52].

In our study, the presence of AP resulted in a reduction in the adhesion of all the tested pathogens. The most highly inhibited strain was *L. monocytogenes* (ATCC 19115), for which there was a reduction in adhesion of 28%. For *E. coli* (ATCC 10536) and *S.* Typhimurium (ATCC 14028), the inhibition rate was between 9.3% and 14.2%, depending on the prebiotic preparation. Ebersbach et al. observed that the addition of xylo-oligosaccharides (XOS) to the pure culture of *L. monocytogenes* resulted in a significant reduction in the adhesion of two of the three tested strains to Caco-2 cells. However, after 2 h of incubation of *L. monocytogenes* sp. with XOS and Caco-2 cells, adhesion was reduced in all three of the tested strains. The presence of other carbohydrates (galacto-oligosaccharides, insulin and polydextrose) did not have such effects. Al-Gazzewi and Tester observed that, of all the sugars they tested, only mannose and glucomannan hydrolysates (GMH) significantly reduced the adhesion of *E. coli* NCTC 8623 to human intestinal epithelium. Parkar et al. (2010) [53] observed that oligosaccharide fractions from kiwifruit affected the adhesion of *Salmonella* sp. and *Lactobacillus* sp. bacteria to Caco-2 cells. The most effective proved to be the soluble fraction monoK, in the presence of which the adhesion of *Salmonella* Typhimurium reduced by 73.7% and an increase of 38% was observed in the adhesion of *Lactobacillus* sp. bacteria. Oligosaccharides were also observed to have anti-adhesive properties against pathogens [54]. The authors found that the pectin oligosaccharides (POS) present in orange peel decreased the adhesion of enteropathogenic *E. coli* and verotoxigenic *E. coli* to HT-29 cells. Furthermore, it was shown that the POS had protective properties against verocytotoxins VT1 and VT2 produced by *E. coli.*

Inhibition of adhesion by pathogens to the intestinal epithelium in the presence of oligosaccharides has also been observed [49,55]. In our study, the adhesion of bacteria isolated from fecal material to Caco-2 cells was less inhibited than that of aerobic bacteria (the inhibition rate was about 3.0% for anaerobes and 7.7%–9.4% for aerobes). In addition, in the presence of preparation P3, containing oligosaccharides with DP 1–6, an increase in adhesion of 2.9% was observed compared to the control. The effect of oligosaccharides on the adhesion of fecal microbiota to the intestinal epithelium has also been studied [56]. In their study, the greatest reduction in adhesion to HT-29 cells was observed in the presence of cellobiose (on average about 65%) and the lowest in the presence of lactulose (approx. 47%). The greatest change in adhesion was observed for *Clostridium* sp. (64%–85%). The lactic acid bacteria/*Enterococcus* sp. and *Bacteroides* sp. were inhibited by 11.7%–58% and 32%–65%, respectively. However, not all the oligosaccharides with antiadhesive properties prevent the development of infections. An example may be FOS, inulin and Xylo-Oligosaccharides. Their prebiotic effect may cause a decrease in resistance to infection by pathogens that cause the invasion of intestinal epithelial cells [57,58]. In summary, the use of certain oligosaccharides affects adhesion to intestinal epithelial cells of both the intestinal microbiota and pathogenic organisms.

## 5. Conclusions

This study set out to evaluate the potential of using apple pomace (AP) for the production of pectin-derived oligosaccharides (POS) by enzymatic and mild acid hydrolysis. In addition, the effects of polymerization of the structural units of POS contained in the AP hydrolysates on the growth and metabolism of microbiota from the human gastrointestinal tract and also on the adhesion of lactic acid bacteria or pathogens to human gut epithelial cells were investigated in vitro. Oligosaccharides with different degrees of polymerization (DP 2–10) were assessed using HPAEC (High-Performance Anion-Exchange Chromatography). Commercial enzymes, cellulases, and pectinases (polygalacturonase, pectin lyase, pectinmethylesterase) were added. The simultaneous use of cellulolytic preparations and pectinase enhanced the yield of oligosaccharides. However, brief acid hydrolysis pretreatment before pectinolysis successfully eliminated the need for cellulase during the later stage of the enzymatic process. Mild acid hydrolysis carried out before pectinolysis with Rohapect Ma PlusT yielded the highest concentration of POS (10.81 g/100 g). In contrast, pure enzymatic processing of the AP performed with a mixed preparation of cellulase and Rohapect Ma PlusT resulted in 1.8-fold lower overall POS. However, the concentration of higher-order oligosaccharides (DP 7–10) was 1.7-fold higher.

The increased ratio of higher-order oligosaccharides caused an increase in the bifidogenic effect as well as affecting the amount and the nature of SCFA produced. Inhibition of *Enterobacteriaceae* was also observed. The POS affected the adhesion of lactic acid bacteria, but this was strongly dependent on the strain. The strongest stimulation of adhesion was observed in the presence of the hydrolysate containing the highest concentration of higher-order oligosaccharides (DP 7–10). Fecal bacteria and the tested pathogens showed much weaker adhesion to the intestinal cells in the presence of the AP hydrolysates. All of the tested POS preparations, containing structurally different oligosaccharides (DPs 2–10 with different ratios of higher order oligosaccharides, as well as DPs 2–6), have the potential to be used as prebiotics for human and animals. They stimulate bowel colonization with lactic acid bacteria and inhibit the development of infections caused by pathogens.

## Figures and Tables

**Table 1:** Selective media used for the cultivation of intestinal microbes and culture conditions.

Microorganism	Cultivation Media	Cultivation Conditions
*Lactobacillus*	Rogosa Agar (MERCK)	37 °C, 6 days, anaerobic
*Bifidobacterium*	Bifidobacterium medium	37 °C, 6 days, anaerobic
*Clostridium*	Tryptose Sulfite Cycloserine Agar (MERCK)	37 °C, 6 days, anaerobic
*Enterococcus*	Bile Aesculin Agar (MERCK)	37 °C, 24 h, aerobic
*Bacteroides*	Agar SCHAEDLERA-KV (MERCK)	37 °C, 6 days, anaerobic
Enterobacteriaceae	Mac Conkey Agar (MERCK)	37 °C, 24 h, aerobic

**Table 2:** Principal factor pattern for different degree of polymerization (DP) type.

	PCA1	PCA2
DP 2	**0.918**	−0.189
DP 3	**0.934**	0.031
DP 4	**0.950**	−0.007
DP 5	**0.967**	−0.035
DP 6	**0.946**	0.246
DP 7	**0.678**	0.593
DP 8	0.109	**0.886**
DP 9	−0.109	**0.955**
DP 10	−0.025	**0.800**

**Table 3:** Stimulation (+) or inhibition (−) rates (%) for adhesion of lactic acid bacteria to Caco-2 cells in the presence of apple pomace preparations: P1, P2, and P3.

Strain	Stimulation (+)/Inhibition Rate (−) (%)		
	P1	P2	P3
*Lb. plantarum* 0981	+10.0	+4.7	+6.0
*Lb. plantarum* 0982	−4.0	+0.9	+1.5
*Lb. brevis* 0983	+13.4	+8.6	+10.7
*Lb. brevis* 0984	−4.1	+4.5	−1.5
*Lb. paracasei* 0985	−10.6	−9.2	−10.4
*Lb. plantarum* 0989	−3.1	−11.3	−5.0
*Lb. plantarum* 0990	−6.0	−9.9	−5.3
*Lb. plantarum* 0991	+0.3	+1.7	−0.9
*Lb. paracasei* 0993	−2.9	+1.2	+1.9
*Lb. plantarum* 0995	+8.7	+3.1	+1.5
*Lb. plantarum* 0996	−0.6	−4.4	−1.8
*Leu. mesenteroides* 0986	−2.9	−0.2	+2.4
*Leu. mesenteroides* 0994	−21.3	−6.8	+2.6

**Table 4:** Adhesion of pathogens and fecal isolates to Caco-2 cells in the presence of apple pomace preparations (P1, P2, and P3). The inhibition/stimulation rate (%) was calculated according to the equation: (adherence of tested sample × 100%/adherence of control) − 100%.

Strain	Inhibition (−)/Stimulation (+) Rate (%)		
	P1	P2	P3
*Escherichia coli* ATCC 10536	−13.9	−12.9	−9.3
*Salmonella* Typhimurium ATCC 14028	−12.0	−13.6	−14.2
*Listeria monocytogenes* ATCC 19115	−27.9	−27.9	−27.9
Aerobic fecal bacteria	−9.4	−7.7	+2.9
Anaerobic fecal bacteria	−2.9	−2.1	−3.1
